# Supplementary material for: Developing Co-Creation Research in Food Retail Environments: A Descriptive Case Study of a Healthy Supermarket Initiative in Regional Victoria, Australia
Source: Int J Environ Res Public Health. 2023 Jun 7;20(12):6077. doi: 10.3390/ijerph20126077 (PMC10298708; doi:10.3390/ijerph20126077)
Supplement: Supplementary file 1 [file ijerph-20-06077-s001.zip › ijerph-2330371-supplementary.pdf]

Supplementary material

Table S1. Focus group/interview guide 2

Table S2 Co-creation frameworks: theoretical underpinning 4

Eat Well Feel Good Ballarat case study description 5

Case study setting 5

Initiation 5

Identification of governance structures and stakeholders 5

Eat Well Feel Good Ballarat strategy development 6

Impacts on store operations during the implementation 8

Evaluation 8

Lessons learned 8

Table S1. Focus group/interview guide.

**Opening/Rapport**		
**Topic**	**Content**	
Self-introduction	Name, background	
Plain language explanation	The objective of the study and aim of the interview	
Record consent for participation	Verbal consent from the participant to be recorded and reiteration of the use of information in a non-identifiable form	
Participant introduction	Understanding the participant's positioning regarding the research topic and experience in food environments research	
**Main interview**		
**Topic**	**Rationale**	**Type of questions**
**Opening question**	Understanding the participant's positioning regarding the research topic and experience in food environments research	• In your own words, what was the *Eat Well Feel Good Ballarat* project about<br>• What was your role in this project/initiative?
**Experiences of the co-creation process**	Explore the perceived factors that positively or negatively influenced this initiative.	• If we split this initiative into steps, what were these steps?<br>• What were the most successful experiences of each of these steps?<br>• What things didn't go as planned?<br>o What did you do? (Resources, staff, training)<br>• Before this project, have you tried any strategy (s) related to healthy eating in-store or in any food retail environment?<br>o What made this project different?<br>o If other stores/groups would do a similar project, what would you recommend?
**Motivation**	What factors motivated stakeholders' participation in the co-creation of strategies to improve the healthiness of food retail outlets?	• What motivated you to be part of this initiative?<br>• Did you learn something from it?<br>o Yes: What?<br>o No: What would you've liked to learn from it?
**Opportunity areas**	Explore the opportunities identified by stakeholders to improve and sustain the process of co-creation	• In terms of collaboration, what would you have done differently?<br>o Is there any relevant experience that could help improve or maintain this process?<br>o How has this process influenced the implementation of *Eat Well Feel Good Ballarat*?<br>• How feasible is this to happen again? – why?
**Structures that enable co-creation**	Discuss the type of structures/policies that facilitate the co-creation of this initiative	• What/who helped you throughout the process of planning, implementing and evaluating?<br>• Did the *Eat Well Feel Good Ballarat* formally impact how healthy food is approached internally?<br>• Would you consider an internal policy?<br>o Why [yes/no]<br>o If yes: What would be needed?
**Future practice**	Explore ideas for future practice/planning	• In your opinion what are the next steps required to improve the healthiness of the store?<br>• Are any other stakeholders that should be involved? Who?<br>• How would you want to be engaged and involved in changing the food environment in the future?
**Closing**		
**Topic**	**Rationale**	

Summary of the interview	Summary of main points and final question to add the missing information
Commitment for transcript	A verbal commitment from the interviewer with a timeframe for returning the transcript

Table S2. Co-creation frameworks: theoretical underpinning.

COACH cycles	Component	Generic co-creation in public health	Component
Stakeholder engagement, evidence collection & Governance	• Challenge identified<br>• Governance & Accountability<br>• Stakeholder engagement<br>• Audit/appraisal	Identify	• Identification of the governance structures and stakeholders relevant to the issue of interest.
		Analyse	• Analysis of the stakeholder network<br>• Identification of processes and options for decision making<br>• Role agreement<br>• Identify relevant experiences and ideas for possible solutions
Communication, policy alignment & development	• Communication & information sharing<br>• Policy & Organisational Policy	Define	• Prioritisation of problems<br>• Next steps and actions
Co-design of evidence-informed action and implementation planning	• Co-design and prioritisation of proposed actions<br>• Implementation & Evaluation Planning<br>• Implementation<br>• Feedback	Design	• Co-design of initiatives by:<br>• Setting goals<br>• Actions to achieve those goals<br>• Evaluation processes<br>• Allocation of resources and assets
		Realise*	• Strategy test<br>• Information gathering
Momentum continuous quality improvement cycle	• Monitoring & Evaluation	Evaluate	• Assessment of:<br>• Proposed outcomes<br>• Previous steps<br>• Learnings from the diverse stakeholders<br>• Changes in the environment<br>• Ways for sustainability

* This realisation stage can remain continuous or occur in stages where testing ideas are re-evaluated.

Eat Well Feel Good Ballarat case study description

Case study setting

The *Eat Well Feel Good Ballarat* (EWFGB) project was conducted with supermarkets in the City of Ballarat, a regional centre located 110 km northwest of Melbourne in Victoria, Australia [1]. It covers an area of 739 square kilometers, and in 2021 Ballarat had a population of 116,201 residents [2]. Ballarat faces a substantial burden from non-communicable diseases. In 2017, 27% of adults had two or more chronic conditions, and on average, 53% of adults had overweight or obesity, with obesity rates higher than state levels [3]. The number of fruits and vegetables consumed by adults in Ballarat was marginally higher than the Victorian average. Still, only 5% met the national fruit and vegetable consumption guidelines, and 45% did not eat enough fruit and vegetables to meet the consumption guidelines [3]. An inadequate diet has also contributed to high rates of cardiovascular disease, type 2 diabetes and some cancers [4, 5].

Initiation

In 2020, Community Health Service (CHS) developed the *Eat Well Feel Good* Ballarat (EWFGB) initiative in response to a community consultation where customers expressed the need for more supermarket support to choose healthier food and drink options [6]. CHS partnered with a Supermarket Chain to pilot the EWFGB initiative. The Independent Grocers of Australia (IGA) is the fourth largest chain of supermarkets in Australia (~1300 stores and 7% market share) [7]. The participant Supermarket is an independent group that started serving the community of Ballarat North in 1979 and has become a prominent chain in this area with six supermarkets [8]. Metcash owns IGA, but individual stores are owned independently.

Identification of governance structures and stakeholders

CHS is the principal organization that has closely partnered with the Supermarket management on the EWFGB project since 2019. The intervention sits within a broader movement of health promotion strategies undertaken in the catchment to promote the creation of supportive environments for healthier eating [9]. Key stakeholders that supported the EWFGB as per Table 1 [10]. Table 1 outlines the stakeholders' involvement and roles in the EWFGB project.

Table 1. Stakeholders' roles and involvement in the *Eat Well Feel Good Ballarat* project.

	Name	Involvement/roles
**Government Institutions**	Community Health Service	• Stakeholder identification<br>• Project management
	Regional Health Service	• Supported the project's inception
	Primary Care Partnership	• Supported the project's inception.
	Victorian State Government Department	• Supported the project's inception.<br>• Provided seed funding for the pilot
	Gov. Funding	• Resources
**Non-Governmental Organizations**	Diabetes focus<br>Nutrition focus<br>Cancer focus	• Resources as a part of the project's campaign materials<br>• Logo endorsement on materials
**Academic Institutions**	Academic Institute	• Assessed products against Health Star Rating criteria.<br>• Provided lists to CHS of packaged products in the Supermarkets that met the rating of 4.5 or 5 stars
	University 1	• Provided access to Store Scout<br>• Assisted in the analysis of Store Scout and sales data.<br>• Placement students (Dietetics Placement)
	University 2	• Provided information regarding the Eat Well @ IGA Bendigo project [11]

		• Supported the pilot, sharing learnings and resources
	University 3	• Placement student (Public Health Placement)
**Supermarket**	Owner	• Supported the trial in three stores
	Managers	• Provided sales data.<br>• Reported any material causality
	Staff	• Supported the implementation
**Community**	Consumers	• Initial consultation and final evaluation
	Volunteers	• Data collection (surveys delivery, in-store follow-up)<br>• Implementation (material delivery and allocation)

Eat Well Feel Good Ballarat strategy development

The project aimed to increase customers' ease of identifying and selecting healthier food and drink products using interventions within the supermarket environment that promote healthier food and drink options. This was carried out using the Health Star Rating (HSR) system (a national front-of-pack labelling system that rates the overall nutritional profile of packaged food and assigns it a rating from ½ a star to 5 stars) [12] and a health promotion campaign. The EWFGB pilot was modelled on critical elements of Eat Well @ IGA [11]. The included strategies were selected in agreement with the store manager. This pilot project utilized promotional materials developed specifically for the Ballarat IGA stores, including shelf wobblers, basket inserts, floor stickers, fridge stickers, banners, recipe cards, staff badges, and staff education materials [10]. Figure 1 shows the EWFGB planning logic model.

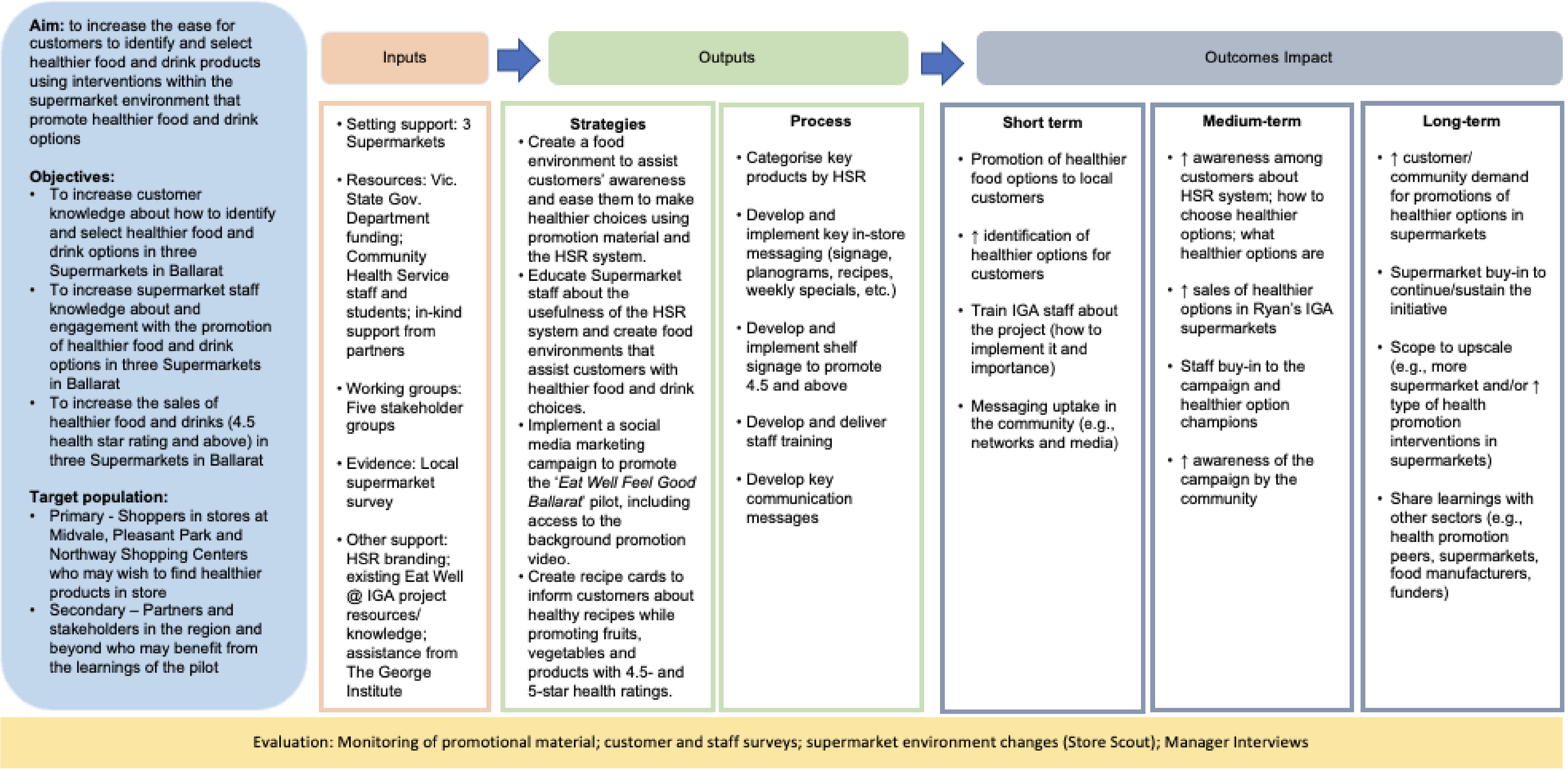

Figure 1. *Eat Well Feel Good Ballarat* trial logic model.

Impacts on store operations during the implementation

EWFGB was piloted in three supermarkets in Ballarat for nine weeks. Before implementation, the supermarket environment was measured with the Store Scout App. This App was designed to provide an immediate appraisal of the store environment by collecting information on the '4Ps' of marketing (Promotion, Price, Product, Placement. It has good reliability and internal consistency in both remote and urban areas, making it an effective tool for communicating health-enabling food retail best-practice with key stakeholders [13]. The implementation had a staggered start (of one week) between stores so that process evaluation could guide each subsequent launch.

Evaluation

The evaluation was designed as a mixed-method evaluation focused on process and impact evaluation. The evaluation included six data collection methods [10, 14]. Table 2 outlines the EWFGB project evaluation.

Table 2. *Eat Well Feel Good Ballarat* project evaluation methods.

Method	Evaluation output
1. Customer exit survey (*n*=259):	• 55% recalled one or more EWFGB project materials.<br>• 43% believed that it influenced them while shopping.<br>• 93% agreed that the supermarket should continue its effort to encourage healthy eating
2. Staff survey (*n*=52):	• 88% showed positive perceptions of the EWFGB project.<br>• 79% believed that the campaign materials did not interfere with their work.<br>• 100% agreed that the Supermarket should continue its effort to encourage healthy eating
3. Semi-structured interviews with IGA staff	• Common themes related to i) the importance of a multi-stakeholder partnership to implement and evaluate the project; ii) continuing the EWFGB initiative.
4. Sales data:	• There was an overall sales increase of 18.6% on healthy food products (breads, frozen fruits, frozen vegetables, and breakfast cereals) at the three supermarkets during the intervention period compared to the baseline
5. The Store Scout App	• Score pre-implementation: 70%<br>• Score post-implementation: 77%
6. In-store monitoring	• Monitoring time: one hour per store per week<br>• Analysis from photographs and template sheets showed that the most resistant materials were the fridge stickers and staff educational materials.<br>• There was no consistency in the placement of banners, and they often were replaced by products in the promotion.<br>• Staff badges were commonly worn by the checkout staff wore the badges.<br>• Recipe Cards were inconsistently placed, and 300 out of 700 recipe cards were taken by shoppers.<br>• The less functional material was the shelf Wobblers. This material was commonly placed in front of the wrong food products, missing or damaged.

Lessons learned

While the store staff's perception of the most noticeable promotional materials were the shelf wobblers, floor stickers and basket inserts, it was considered that the shelf wobblers sometimes covered the price tags. The perception on components that did not work well were fridge stickers and badges. The staff room can be considered an ideal setting to provide employees with information about the initiative. Yet, there is the opportunity to increase the type of resources and other actions for staff awareness of the initiative [10].

Working in partnership was the main enabler in planning, implementing, and evaluating the initiative. Acknowledging the Supermarket experience and the open and continual communication between the Supermarket and CHS was critical for a successful implementation. It was considered that CHS leadership gave higher credibility to the EWFGB trial as the leading association of this stakeholder is to a health service instead of the food industry [10].

One of the main challenges was the impact COVID-19 had on the implementation. Lockdown restrictions during the EWFGB trial cause customers to spend less time in the supermarkets. Moreover, QR codes for customer survey challenges were redirected to fewer traffic areas to accommodate the contact tracing QR codes. Consequently, the survey had less participation than expected [10].

References:

1. Regional Development Victoria. Regional City of Ballarat. Availabe online: https://www.rdv.vic.gov.au/victorias-regions/ballarat (accessed on 30 August 2022).
2. Australian Bureau of Statistics (ABS). 2021 Census All Persons QuickStats: Ballarat. Availabe online: https://www.abs.gov.au/census/find-census-data/quickstats/2021/20101 (accessed on 30 August 2022).
3. Department of Health and Human Services. Victorian Population Health Survey 2017. Availabe online: https://www.health.vic.gov.au/population-health-systems/victorian-population-health-survey-2017 (accessed on 30 August 2022).
4. City of Ballarat. Health and Wellbeing Plan 2021–2031. Availabe online: https://www.ballarat.vic.gov.au/sites/default/files/2021-10/Health%20and%20Wellbeing%202021-2031_LR.pdf (accessed on 30 August 2022).
5. Central Highlands Primary Care Partnership. City of Ballarat: Health and Wellbeing snapshot. Availabe online: https://vicpcp.org.au/wp-content/uploads/2022/06/LGA-Data-Snapshot-Ballarat-C-2022.pdf (accessed on 30 August 2022).
6. Greenslade, D.; Ferry, L. *Making Healthier Food Front and Center: How Supermarkets Help or Hinder (Unpublished)*; Ballarat Community Health: Ballarat, Victoria, Australia, 2019.
7. Statista. Market share of grocery retailers in Australia in 2021. Available online: https://www.statista.com/statistics/994601/grocery-retailer-market-share-australia/ (accessed on 15 March 2022).
8. Ryan’s IGA. About us. Availabe online: https://ryansiga.com.au/about-us/ (accessed on 30 August 2022).
9. Ballarat Community Health and Central Highlands Primary Care Partnership. Healthy Lifestyle Programs. Availabe online: http://www.chpcp.org/wp-content/uploads/2014/11/Healthy-Lifestyle-Programs_HP-version.pdf (accessed on 30 August 2022).
10. Thuruthikattu, F.; Deutscher, M.; Greenslade, D. *Eat Well Feel Good Ballarat: Evaluation Report (Unpublished)*; Ballarat Community Health: Ballarat, Victoria, Australia, 2021.
11. Blake, M.R.; Sacks, G.; Marshall, J.; Brown, A.K.; Cameron, A.J. A successful Intervention research collaboration between a supermarket chain, the local Government, a Non-governmental Organization and academic researchers: The Eat Well @ IGA Healthy Supermarket Partnership. In *Global Handbook of Health Promotion Research, Vol. 1: Mapping Health Promotion Research*; Potvin, L., Jourdan, D., Eds.; Springer: Cham, Switzerland, 2022; pp. 343–364. https://doi.org/10.1007/978-3-030-97212-7_24.
12. Commonwealth of Australia. Health Star Rating System. Availabe online: http://www.healthstarrating.gov.au/internet/healthstarrating/publishing.nsf/Content/Home (accessed on 31 August 2022).
13. McMahon, E.J.; Jaenke, R.; Brimblecombe, J. A Mobile App to Rapidly Appraise the In-Store Food Environment: Reliability, Utility, and Construct Validity Study. *JMIR Mhealth Uhealth*. **2020**, *8*, e16971.
14. McWilliam, L.; Darling, G. *Eat Well Feel Good Ballarat' Healthy Supermarkets Project: Evaluation Plan (Unpublished)*; Community Health Service: Ballarat, Victoria, Australia, 2021.
